# Development of an effective one-step double-antigen sandwich ELISA based on p72 to detect antibodies against African swine fever virus

**DOI:** 10.3389/fvets.2023.1160583

**Published:** 2023-06-09

**Authors:** Lei Wang, Duan Li, Yanlin Liu, Leyi Zhang, Guoliang Peng, Zheng Xu, Hong Jia, Changxu Song

**Affiliations:** ^1^College of Animal Science and National Engineering Center for Swine Breeding Industry, South China Agricultural University, Guangzhou, China; ^2^Henry Fok School of Biology and Agriculture, Shaoguan University, Shaoguan, China; ^3^Institute of Animal Sciences, Chinese Academy of Agricultural Sciences, Beijing, China

**Keywords:** African swine fever virus, p72 protein, one-step ELISA, double antigen sandwich ELISA, antibody detection

## Abstract

African swine fever (ASF), caused by ASF virus (ASFV), is a highly contagious and lethal disease of domestic pigs leading to tremendous economic losses. As there are no vaccines and drugs available. An effective diagnosis to eliminate ASFV-infected pigs is a crucial strategy to prevent and control ASF. To this end, ASFV capsid protein p72 was expressed using Chinese hamster ovary (CHO) cells and subsequently conjugated with horseradish peroxidase (HRP) to develop a one-step double-antigen sandwich enzyme-linked immunosorbent assay (one-step DAgS-ELISA). The performance of this ELISA for detecting ASFV antibodies was evaluated. Overall, a diagnostic sensitivity of 97.96% and specificity of 98.96% was achieved when the cutoff value was set to 0.25. No cross-reaction with healthy pig serum and other swine viruses was observed. The coefficients of variation of the intra-assay and inter-assay were both <10%. Importantly, this ELISA could detect antibodies in standard serum with 12,800-fold dilution, and seroconversion started from the 7th day post-inoculation (dpi), showing excellent analytical sensitivity and great utility. Furthermore, compared to the commercial kit, this ELISA had a good agreement and significantly shorter operation time. Collectively, a novel one-step DAgS-ELISA for detecting antibodies against ASFV is developed, which will be reliable and convenient to monitor ASFV infection.

## 1. Introduction

African swine fever (ASF) is a highly contagious and lethal swine disease, which causes severe socioeconomic losses. It has been listed as a notifiable animal disease by the World Organization for Animal Health (OIE) in 2004 (1). ASF was first reported in Kenya in 1921 (2). Since then, it has been rapidly spread and epidemic in 60 countries across Europe, Latin America, and Asia (3). ASF was first reported in China in 2018 and brings tremendous economic losses to China’s swine industry, similarly (4). Unfortunately, there are no vaccines or effective treatments to control ASF worldwide (5). Effective approaches to control and prevent ASF include early diagnosis, and rapid elimination of infected pigs (6). Therefore, a sensitive and reliable diagnostic assay is crucial for ASF control.

ASF is caused by ASF virus (ASFV). In the absence of ASFV vaccination, the presence of ASFV antibodies indicates historic infection. Serological tests have been introduced into ASF control and eradication programs. Commercial ELISAs have been stipulated by OIE (7). According to different principles, ELISA is developed into various types, including direct ELISA, indirect ELISA, sandwich ELISA, competitive ELISA, and so on (8). However, there time-consuming operation and high-cost are major drawbacks. This study aims to develop a novel double-antigen sandwich enzyme-linked immunosorbent assay (DAgS-ELISA) in hopes of realizing more simple, sensitive, and accuracy detection of ASFV antibodies. This ELISA is established by utilizing HRP-conjugated antigen, then one-step incubation of HRP-conjugated antigen and serum, for the first time.

ASFV is a large, double-stranded DNA virus which belongs to the *Asfarviridae* family (9). The genome size of ASFV is about 170–194 kb, and encodes more than 150 viral proteins (10). ASFV virion has a complex structure with multiple membrane and protein layers. To date, 68 virion proteins have been identified, among them, p72 (B646L) with a molecular weight of 73.2 kDa, is the most predominant virion protein and comprises 31 ~ 33% of the virion protein mass (11). Moreover, p72 is a high immunogenic viral protein, and well suited as a promise candidate for diagnostic purpose (12). Many diagnostic methods have been established based on p72, and show excellent performance, like lateral flow assay (13). In fact, ELISA is more widely used due to its advantage of high-throughput qualitative and quantitative analysis.

Therefore, in this study, we successfully obtained ASFV p72 and innovatively established a one-step DAgS-ELISA to detect ASFV antibodies. This ELISA showed sensitivity and rapid for assessment of ASFV infection, which will be a potent detection tool for clinical application to control ASF.

## 2. Materials and methods

### 2.1. Serum standard and testing samples

All sample treatments were strictly performed in accordance with the standard operation for ASFV by OIE. The ASFV-positive standard serum and the ASFV-positive standard serum were obtained from the China Veterinary Drug Administration. The serum of Swine immunized with ASF gene-deleted vaccine candidate (at 0, 7, 14, 21, and 28 dpi) were provided by the program team of African swine fever gene-deleted vaccine research and development of the National Key R&D Plan program of China. All 344 sera including 283 negative sera collected before the outbreak of ASFV in China from 2016 to 2017 and 61 positive sera collected from clinically infected pigs were confirmed by gold standard and stored in our laboratory.

Healthy swine negative sera, positive sera against pathogenic classical swine fever virus (CSFV), pseudorabies virus (PRV), porcine circovirus type 2 (PCV2), porcine reproductive and respiratory syndrome virus (PRRSV), foot-and-mouth disease virus (FMDV) and porcine epidemic diarrhea virus (PEDV) were identified and stored in our laboratory.

### 2.2. Expression, purification and conjugation of ASFV-P72

The DNA fragment of ASFV B646L gene (GenBank: QBH90570.1) was codon-optimized and cloned into pCAGGS-His vector. The plasmids expressing p72 was transfected to CHO cells using TransIntro^®^ EL/PL Transfection Reagent (No FT231-02, TrasnGen Biotech, Beijing, China). After 3–4 days post transfection, the cells were harvested and resuspended in phosphate-buffered saline (PBS, pH 7.5) containing protease inhibitor cocktail (No539133, Solarbio, Beijing, China). The soluble p72 was purified by BeaverBeads™ IDA-Nickel Kit-10 (No70501-K10, Beaver, Suzhou, China). The purified sample was subjected to gel filtration chromatography with HiLoad^®^ 16/600 Superdex^®^ 200 pg (No 28989335, GE Healthcare, Uppsala, Sweden). The eluted sample was analyzed by in sodium dodecyl sulfate polyacrylamide gel electrophoresis (SDS-PAGE), followed by immunoblotting (IB) using ASFV-positive standard serum. The soluble p72 was dissolved in PBS (pH 7.5), at a concentration of 1 mg/ml, and coupled to HRP using a Lightning-Link HRP conjugation kit (No701-0010; Innova Biosciences, Cambridge, United Kingdom) according to the instructions.

### 2.3. Establishment and optimization of one-step DAgS-ELISA

The working concentrations of the soluble p72 and serum were determined by checkerboard titration according to a previous study with some modification (14). Briefly, the recombinant p72 was diluted at 0.15, 0.3, 0.6, and 1.2 μg/ml. The ASFV positive and negative standard sera were diluted at 1:1, 1:5, 1:10, 1:20, and 1:50. The HRP-conjugated p72 was used at 1:6,000. After that, the 96-well ELISA plates (Corning, New York, United States) were coated with the diluted p72 in 0.05 M carbonate–bicarbonate buffer (pH 9.6) at 4°C for 12 h, and then blocked with 5% bovine serum albumin (BSA) at 37°C for 1 h. After three washes, the plates were co-incubated with 50 μl of diluted HRP-conjugated p72 and 50 μl of diluted sera at 37°C for 1 h. The plates were washed and added with 100 μl of 3,3′,5,5′-tetramethylbenzidine (TMB) at 37°C for 15 min in the dark, the reaction was stopped using 50 μl of 0.3 M H_2_SO_4_. The optical density (OD) values were measured at 450 nm using an ELISA microplate reader (BioTek, VT, United States). All samples are tested in duplicate. The OD_450_ ratio between positive and negative sample (P/N value) was calculated.

Next, in order to optimize working condition, the OD450 value and P/N value of different conditions were compared. Different coating condition (37°C 2 h, 37°C 1 h, 4°C 6 h, 4°C 12 h) were explored. Different blocking solutions (5% skim milk, 1% casein, and 5% BSA) and blocking condition (0.5, 1, 2, 3 h) were explored, respectively. The TMB response time (5, 10, 15 min) was optimized. The HRP-conjugated p72 was diluted at 1:2,000, 1:4,000, 1:6,000, 1:8,000 and 1:10,000, and tested. The optimal reaction time of serum and HRP-conjugated p72 (20, 30, 40, 60 min) was also tested. The maximum P/N value was scored as optimal working conditions.

### 2.4. Cut-off value, diagnostic sensitivity and specificity determination

To calculate the optimal cutoff value, and associated diagnostic sensitivity and specificity, a total of 344 serum samples including 283 negative samples and 61 positive samples from individual pigs were tested by one-step DAgS-ELISA. Receiver operating curve (ROC) analysis was performed by MedCalc software (15). The sensitivity and specificity of the established ELISA were calculated.

### 2.5. Cross-reactivity detection

ASFV-positive and negative sera as well as CSFV, PRRSV, PCV2, PRV, FMDV-positive sera were tested by the established one-step DAgS-ELISA.

### 2.6. Reproducibility assessment

Intra and inter-assay variation were evaluated according to a previous study with some modifications (16). Briefly, eight serum samples (three negative control, three medium-positive control, and two strong-positive control) were randomly selected. Three replicates of each sample were assayed in one batch to evaluate intra-assay (within plate) variation and three plates were assayed as separate batches to evaluate inter-assay (between assays) variation. Means, standard deviations, and percent coefficient of variation (CV) were calculated.

### 2.7. Detection antibody in ASFV-immunized pig sera, ASFV positive standard serum

The serum of swine immunized with ASF gene-deleted vaccine candidate at different time-points (0, 7, 14, 21, and 28 dpi; *n* = 5/each stage) were diluted at 1:10. ASFV-positive and negative standard serum were subjected to two-fold dilutions from 1:50 to 1:21,600, respectively. All diluted serum samples were tested by the established one-step DAgS-ELISA.

### 2.8. Comparison with commercial kits

All 344 sera including 283 negative sera and 61 positive sera were tested using one-step DAgS-ELISA and two commercial ELISA kits: blocking ELISA (Ingenasa, Madrid, Spain), and competition ELISA (ID.vet, Grabels, France) (3, 16). Results were compared by calculating the concordance rate to evaluate the performance of one-step DAgS-ELISA.

### 2.9. Statistical analysis

Statistical analysis was conducted by using GraphPad Prism version 8.0 software (San Diego, CA, United States). The cutoff value was determined by ROC curve. All data are shown as means ± standard deviations (SD). The reproducibility was evaluated by the CV.

## 3. Results

### 3.1. Production of p72

**Figure 1:**
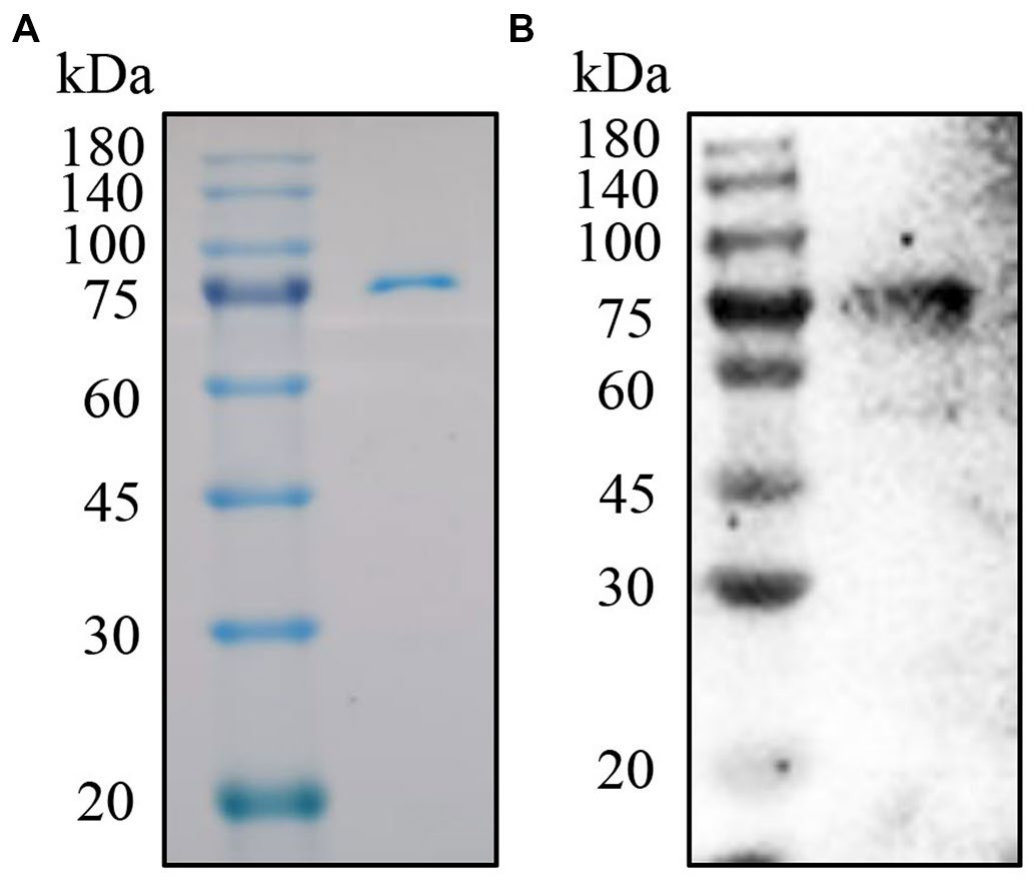
Purification and analysis of the p72. **(A)** SDS-PAGE analysis of p72 by anti-His tag beads. **(B)** IB analysis of p72 with ASFV positive standard serum.

In order to obtain soluble p72 with high quality. We expressed ASFV p72 in CHO cells. The soluble p72 was then purified by his-tag beads and further eluted by gel filtration chromatograph. The eluted p72 was analyzed by SDS-PAGE and IB. As shown in Figures 1A,B, Coomassie blue staining showed a sharp band around 75 kDa with high purity. While, IB demonstrated a good antigenicity of the soluble p72 against ASFV-positive standard serum.

### 3.2. Establishment of one-step DAgS-ELISA using conjugated p72

**Figure 2:**
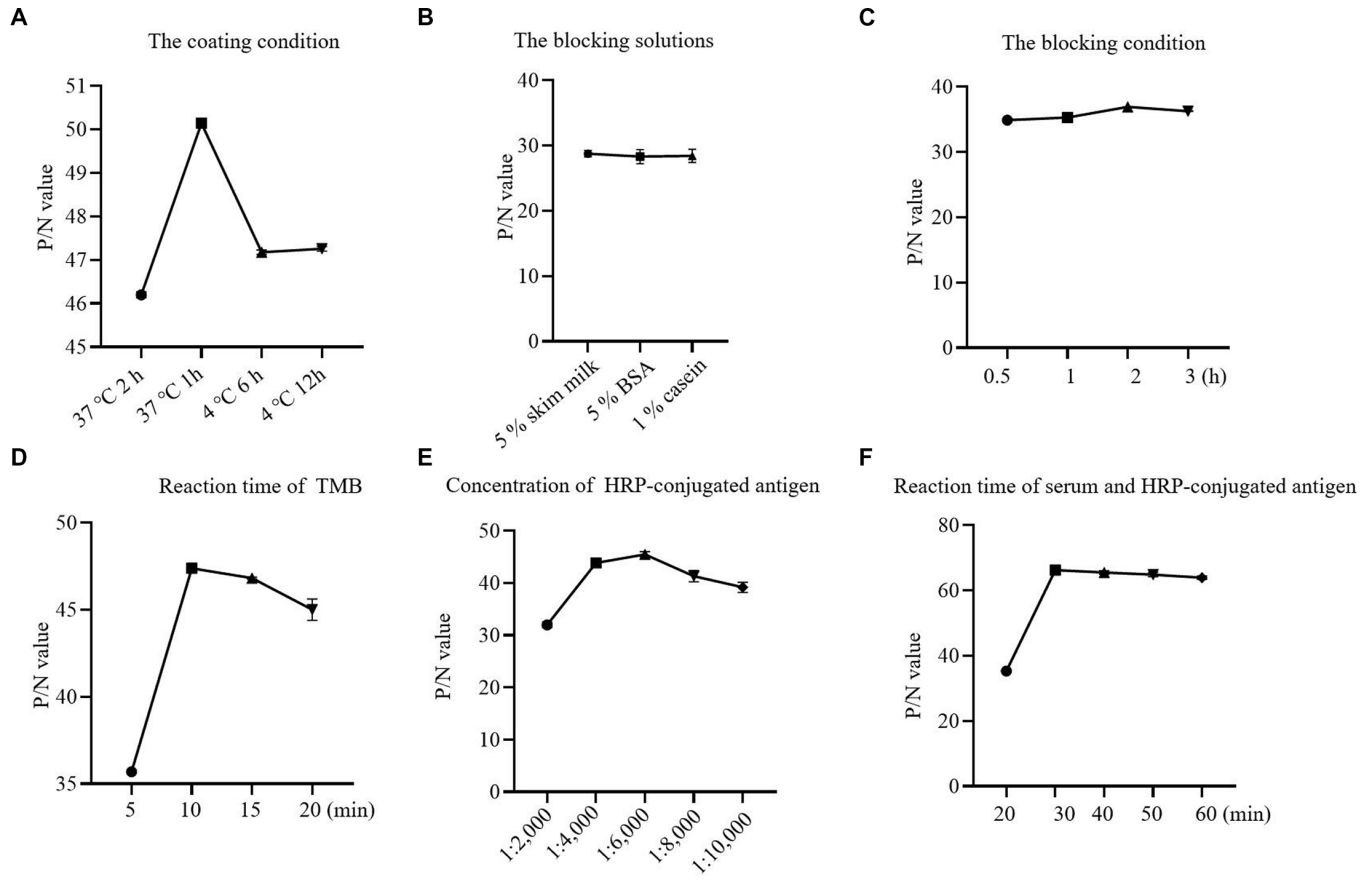
Optimization results for one-step DAgS-ELISA procedure. **(A)** Determination of optimal coating conditions. **(B)** Determination of optimal blocking solutions. **(C)** Determination of optimal blocking conditions. **(D)** Optimal incubation time for TMB. **(E)** Optimal concentration for HRP-labeled p72. **(F)** Optimal incubation time for serum and HRP-labeled p72.

Checkerboard titration was applied to investigate the concentration of the coating antigen and sera. The maximum P/N (55.78) value was obtained when the concentration of p72 was 1.2 μg/ml, the dilution of serum was 1:10 (Table 1). At the same time, the reaction temperature, time, and other conditions were optimized by the index of the P/N value. In brief, the optimum coating condition was 1 h at 37°C (Figure 2A). The blocking solution was selected as 5% skimmed milk in PBST, in this study, three types of blocking solution displayed similar performance (Figure 2B). the optimum blocking condition was 2 h at 37°C (Figure 2C). Furthermore, the optimal reaction time for TMB was 10 min (Figure 2D). The concentration of HRP-conjugated p72 was diluted at 1:6,000 (Figure 2E). Finally, the optimal reaction time for serum and HRP-conjugated p72 was 30 min (Figure 2F). To this extent, the one-step DAgS-ELISA was established.

**Table 1:** Results of P/N value at different conditions.

Dilution of sera		Antigen at different concentration (μg/mL)			
		0.15	0.3	0.6	1.2
	P	0.23	0.51	1.27	2.19
1:1	N	0.05	0.05	0.05	0.05
	P/N	4.80	11.14	27.78	45.52
	P	0.28	0.64	1.41	2.33
1:5	N	0.04	0.04	0.09	0.05
	P/N	6.28	14.40	15.47	44.88
	P	0.33	0.76	1.71	2.44
1:10	N	0.04	0.05	0.05	0.04
	P/N	7.28	16.41	35.49	55.78
	P	0.39	0.88	1.78	2.30
1:20	N	0.05	0.05	0.04	0.04
	P/N	8.46	19.36	40.19	54.76
	P	0.32	0.74	1.44	2.06
1:50	N	0.05	0.05	0.04	0.04
	P/N	6.86	15.12	31.96	45.78

P: OD value of positive samples; P: OD value of positive samples.

### 3.3. Cut-off value, diagnostic sensitivity and specificity determination for one-step DAgS-ELISA

**Figure 3:**
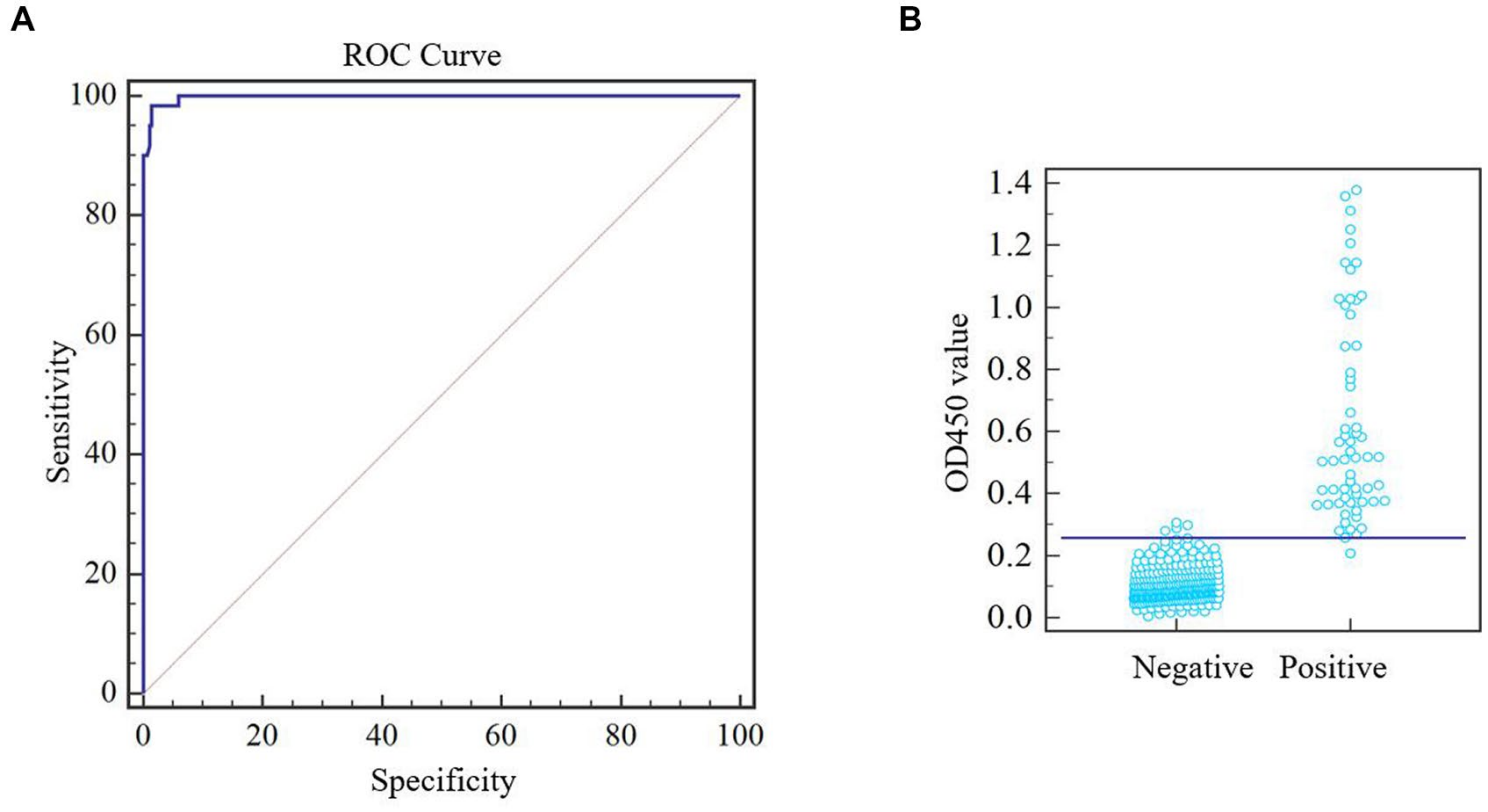
ROC analysis of one-step DAgS-ELISA. ASFV-negative samples (*n* = 283) and ASFV-positive samples (*n* = 61) were measured. Meta-analysis was performed using MedCalc software. **(A)** ROC cure diagram. **(B)** Interactive dot plot diagram.

After optimizing the protocol for assay, a total of 344 pig serum samples (281 negative samples and 63 positive samples) were tested in duplicate by the one-step DAgS-ELISA. Data were entered into the MedCalc software and ROC curve statistical analysis was performed to calculate the cutoff value and draw an interactive dot plot diagram. As shown in Figures 3A,B, the area under the curve (AUC) was 0.996 based on the ROC analysis, as an AUC value above 0.9 indicates excellent diagnostic accuracy (17). Besides, a diagnostic sensitivity of 97.96% and an diagnostic specificity of 98.96% were achieved, when the cutoff value was set to 0.25. These results demonstrated excellent accuracy of the established ELISA.

### 3.4. Repeatability of one-step DAgS-ELISA

The intra-batch variation and inter-batch variation were used to validate the repeatability of the one-step DAgS-ELISA. The CV of both the intra-assay and inter-assay were lower than 10% (Table 2). These results indicated that the newly ELISA has high repeatability and low variability.

**Table 2:** Results of the repeatability assay for one-step DAgS-ELISA.

Sample number	Inter-assay CV (%)		Intra-assay CV (%)	
	M ± SD	CV%	M ± SD	CV%
1	0.093 ± 0.013	3.21	0.083 ± 0.023	6.31
2	0.105 ± 0.042	3.14	0.115 ± 0.032	4.24
3	0.268 ± 0.026	4.52	0.308 ± 0.036	5.42
4	0.312 ± 0.042	3.74	0.342 ± 0.032	4.04
5	0.693 ± 0.053	1.62	0.653 ± 0.043	2.52
6	0.805 ± 0.022	4.25	0.825 ± 0.032	5.38
7	1.468 ± 0.016	2.38	1.508 ± 0.026	3.42
8	1.521 ± 0.022	5.12	1.431 ± 0.042	6.18

### 3.5. Cross-reactivity - and analytical sensitivity of one-step DAgS-ELISA

**Figure 4:**
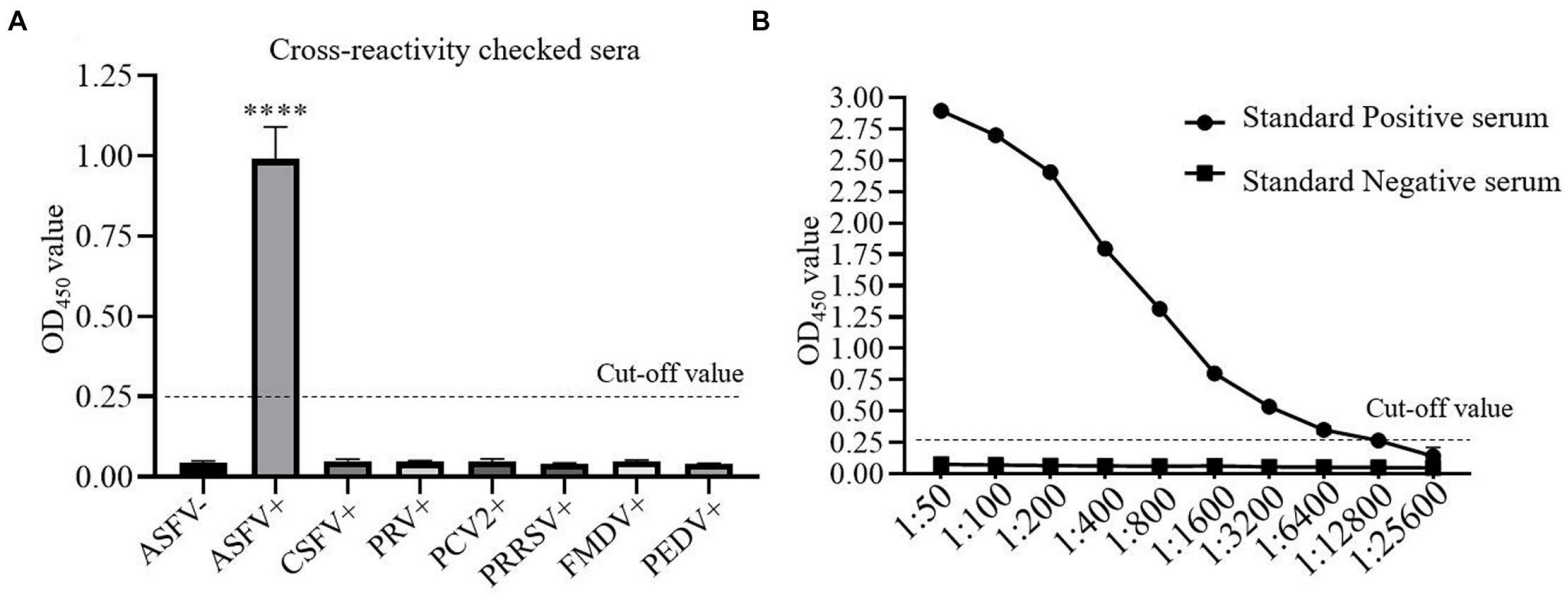
The cross-reactivity and analytical sensitivity test of one-step DAgS-ELISA. **(A)** Specificity assay. ASFV-positive serum, ASFV-negative serum and CSFV, PRV, PCV2, PRRSV, FMDV and PEDV-positive serum were measured. Data represent means ±SD from three independent experiments. *p* < 0.001, ****. **(B)** Sensitivity assay. The ASFV-positive and negative standard serum at different dilutions were titrated with 2-fold dilutions from 1:50 to 1:25,600. The dashed line represents the cut-off of one-step DAgS-ELISA.

The cross-reactivity of one-step DAgS-ELISA was evaluated by testing ASFV-positive serum, negative serum, and serum positive for CSFV, PRV, PCV2, PRRSV, FMDV and PEDV. All serum samples with 10-fold dilution were applied to the one-step DAgS-ELISA. As shown in Figure 4A, only OD_450_ value of ASFV-positive serum was above 0.25, which indicates that the established ELISA has high specificity for detection of ASFV antibodies.

The detection limit of the simplified ELISA was evaluated by two-fold serially diluted ASFV-positive standard serum. As shown in Figure 4B, as the OD_450_ value at the test above 0.25 is considered positive, the detection limit of the established ELISA reached up to 1:12,800, which indicates that the established ELISA has high sensitivity for detection of ASFV antibodies.

### 3.6. Antibody response to p72 in ASFV gene-deleted vaccine candidate-immunized pigs

**Figure 5:**
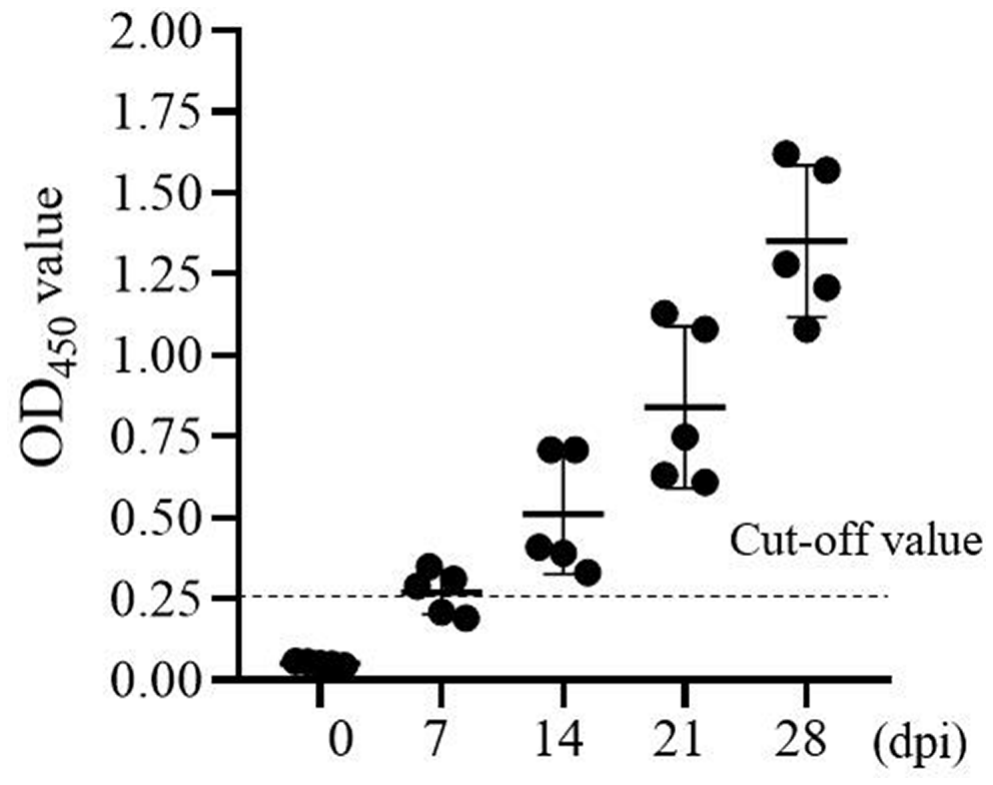
Kinetics of antibody response of swine immunized with ASF gene-deleted vaccine candidate (at 0, 7, 14, 21, and 28 dpi), the dashed line represents the cut-off.

Next, The ASF gene-deleted vaccine candidate-immunized antibody response to ASFV was determined using one-step DAgS-ELISA. Five samples of each stage were obtained. As shown in Figure 5, the antibody response against p72 was detected as early as 7 days after vaccination in three out of five pigs, and all seroconversion around 14 dpi. Since then, the level of antibodies continued to increase in all pigs until 28th day. These results showed that the one-step DAgS-ELISA based on p72 also has the potential to evaluate immune effectiveness.

### 3.7. Evaluation of one-step DAgS-IELISA in comparison with commercial kits

To evaluate the clinical application performance of the one-step DAgS-ELISA, 344 serum samples including 283 positive samples and 61 negative samples were also tested by two commercial ELISA kits. As shown in Table 3, the established ELISA had good performance showing by the total coincidence rate with ID.vet’ ELISA was 96.80%, and Ingenasa’ ELISA was 95.06%, respectively. While, when detecting positive sample, the coincidence rate between the newly ELISA and ID.vet’ ELISA was 88.52%, and Ingenasa’ ELISA was 81.97%, respectively. To our surprise, three different methods displayed excellent agreement when assessing negative samples, the coincidence rate among them were about 98%. These results suggested that the one-step DAgS-ELISA has high sensitivity and is a more reliable application for detection of ASFV antibodies.

**Table 3:** Comparison of one-step DAgS-ELISA with commercial kits.

	One-step DAgS-ELSIA		
	Positive CR	Negative CR	Total CR
ID.vet ELISA	88.52% (54/61)	98.59% (279/283)	96.80% (333/344)
Ingenasa ELISA	81.97% (50/61)	97.88% (277/283)	95.06% (327/344)

CR, coincidence rate.

## 4. Discussion

ASFV causes a serious economic impact around the world especially in China which has the largest pig industry (5, 18). Currently, no commercial vaccine or drugs is available for ASFV. The principal strategy for ASF control remains early detection and quarantine of affected herds. Although, polymerase chain reaction (PCR), real-time PCR, and ELISA are all recommended by OIE (19). The presence of ASFV attenuated variants makes nucleic acid diagnosis more difficult. Hence, an effective ELISA for detection of ASFV antibodies is of great significance for ASF prevention and control (20).

**Figure 6:**
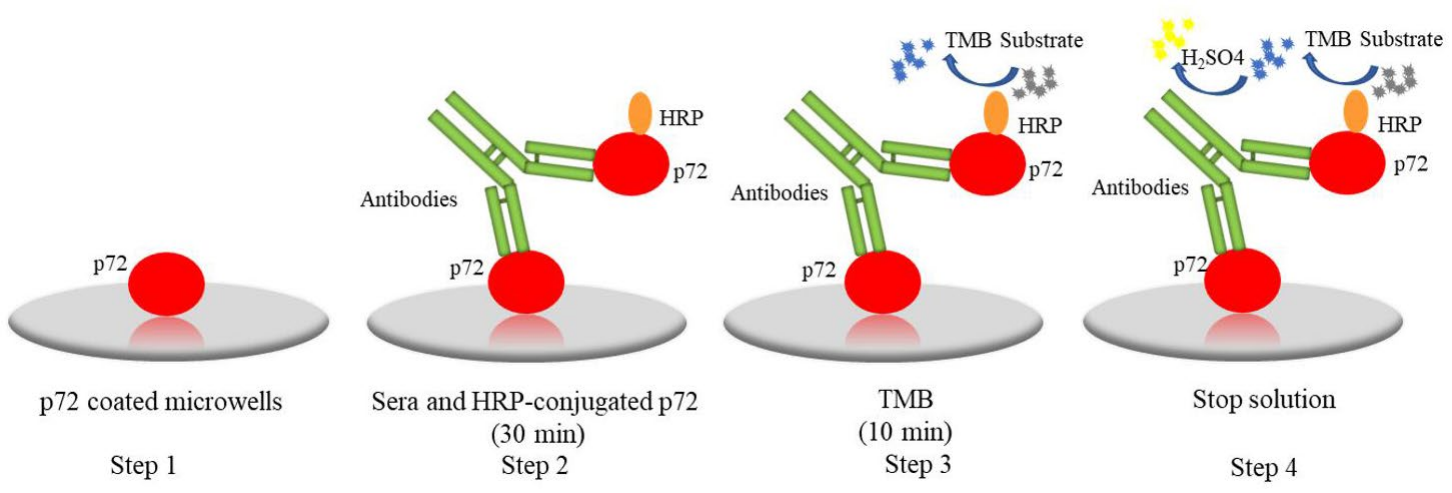
The procedure of one-step DAgS-ELISA: step1, the p72 were coated on plates; step 2, serum and HRP-conjugated p72 were co-incubated for 30 min; step 3, the TMB was added and incubated for 10 min; step 4, the stop solution was added and the OD_450_ value was measured.

In practical application, like Ingenasa from Spain and ID. vet from France, etc., have been widely used in clinical sample testing and play important roles in ASF control. However, non-specific rection, time-consuming, and costly problem impels us to develop novel diagnostic method. This study, a novel one-step DAgS-ELISA was developed with great advantage. As shown in Figure 6. The newly ELISA was established based on double antigen sandwich ELISA (DAgS-ELISA). The DAgS-ELISA was further optimized with one-step incubation of samples and HRP-conjugated antigen. The DAgS-ELISA can detect various types of antibodies in the sample, including IgM, IgA and IgG, and displays higher specificity and better sensitivity, due to the usage of HRP-conjugated antigen (21, 22). The DAgS-ELISA has been applicated for human and animal diseases in pandemics, like severe acute respiratory syndrome-associated coronavirus (SARS-CoV), Chikungunya virus (CHIKV), and ASFV (23–25). Compared with commercial kits, the newly established ELISA has been simplified. Its results can be obtained within 60 min. To our knowledge, this method is the first attempt to be applied to the detection of ASF. Wile, the simplified procedure has been applied for human T-cell lymphoma/leukemia virus type-1 (HTLV-1) antibodies with high accuracy and specificity (26).

Several principal serological immunodeterminants of ASFV have been identified, such as p30, p54, p72, CD2v, pB602L, and K205R, etc., (27). Among them, p72 is considered as suitable candidates for ASFV serological tests. According to previous studies, preparation of soluble p72 is the key for antibody test (28). Correspondingly, the soluble p72 was successfully expressed in CHO cells (Figure 1). The soluble protein was purified and used in serological tests. Currently, many different methods using p72 have been established to detect antibody or antigen. For example, Wang et al. established a blocking ELISA to detect ASFV antibodies (7). Gen et al. and Zhu et al. developed colloidal gold immunochromatographic test strip to detect ASFV antibodies (29). While, Chen et al. developed an alphaLISA to detect ASFV in porcine serum (30). These results together, reflected the application potential of p72 in pandemics.

Next, this one-step DAgS-ELISA based on p72 displayed good diagnostic sensitivity of 97.96% and specificity of 98.96%, when the cutoff value was selected at 0.25 (Figure 3), and high specific to ASFV-positive serum, without cross-reactivity to other swine virus positive sera (Figure 4). The CV in intra-assay and inter-assay repeatability test were all less than 10% (Table 2). Importantly, this one-step DAgS-ELISA was able to detect seroconversion at 7 dpi and ASFV-positive standard sera at a maximum dilution of 1:12800 (Figures 4, 5). The concordance rate with ID.vet’ ELISA was 96.80% (Table 3). As ID.vet’ ELISA has a relatively high sensitivity and better performance among kits from ID.vet, Ingenasa, and Svanova (31). In consistent with the previous study, Zhu et al., described a double-antigen-sandwich lateral-flow assay based on p72, which can also detect seroconversion from 7 to 9 dpi (13). As, the presence of antibodies against ASFV from 7 to 10 dpi (28). Considering good performance, high-throughput qualitative and quantitative detection, simplified operation progress, and low cost, this ELISA is of great significance for the prevention, control, and eradication of ASF.

## 5. Conclusion

This study, a novel one-step DAgS-ELISA for ASFV antibody detection was successfully established. It has good reproducibility and specificity, and production and operation are simple. This study lays the foundation for large-scale detection of ASFV antibody to control ASF, and provides a reference for the detection of other infectious diseases.

## Data availability statement

The original contributions presented in the study are included in the article/supplementary material, further inquiries can be directed to the corresponding authors.

## Author contributions

LW and DL: formal analysis, investigation, validation, writing – original draft. GP: conceptualization, formal analysis, methodology. CS and HJ: conceptualization, funding acquisition, and project administration, manuscript revised. All authors contributed to the article and approved the submitted version.

## Funding

The work was supported by grants from the National Key R&D Program of China (2021YFD1801200 and 2021YFD1801205).

## Conflict of interest

The authors declare that the research was conducted in the absence of any commercial or financial relationships that could be construed as a potential conflict of interest.

## Publisher’s note

All claims expressed in this article are solely those of the authors and do not necessarily represent those of their affiliated organizations, or those of the publisher, the editors and the reviewers. Any product that may be evaluated in this article, or claim that may be made by its manufacturer, is not guaranteed or endorsed by the publisher.
